# Supplementary figures and images for: Metal Oxide Nanorods-Based Sensor Array for Selective Detection of Biomarker Gases
Source: Sensors (Basel). 2021 Mar 9;21(5):1922. doi: 10.3390/s21051922 (PMC7967152; doi:10.3390/s21051922)

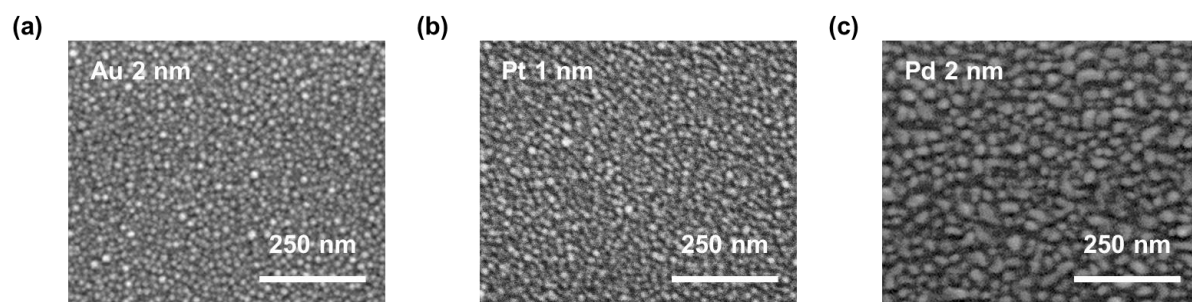

**Figure S1.** Top-view FE-SEM images of (a) Au 2 nm, (b) Pt 1 nm, and (c) Pd 2 nm on SiO<sub>2</sub> substrate.

Supplement: Supplementary file 1 [file sensors-21-01922-s001.pdf]
